# The Montreal Veterinary Medical Association

**Published:** 1893-03

**Authors:** 


*Montreal Veterinary Medical Association.*—The regular meeting of the Montreal Veterinary Medical Association was held in the lecture theatre of the faculty building, 6 and 8 Union Avenue, December 21st, 1892, the Vice-President, Dr. C. McEachran, in the chair.

The business of the evening finished, Mr. Brainard was called upon to report a case of "artificial impregnation in a mare."

The mare was a black roadster, fifteen hands high and eighteen years old, and was what is commonly known as a "constant horser." Her owner had failed to ever get her in foal, she having been served more than fifty times within the last five years.

On examination, the os was found to be hard and contracted, but managed to dilate sufficiently to allow the little finger to be inserted.

Another mare was served, and a small, wide-mouthed bottle, containing water at about blood heat, was inserted into her vagina, and a small quantity of semen collected. This bottle and its contents was passed into the vagina of the subject, and inverted over the mouth of the uterus. The mare was then taken home, became quiet, laid on flesh, and at the end of the usual term gave birth to a healthy, active foal.

After some discussion on this interesting case, Mr. Campbell, the essayist of the evening, was called upon for his paper on "Diet for the Dog." This essay was carefully prepared, and gave rise to much discussion. He commenced by giving the definition of the word "diet." It was derived from the Greek word διαιτα, meaning a system or mode of living.

The body is composed essentially of a semi-fluid substance called protoplasm, whose chemical constituents were C. H. O. N. P. K. Na. Mg. Cl., etc., combined in a very complex molecule, containing water. This protoplasm is the basis of life, and is constantly wasting away by a process of oxidation, and is being as constantly built up by a fluid in the blood which was in turn prepared especially for this complex substance.

As the name of the essay implies, the materials from which protoplasm is built up will be more particularly dealt with, *i. e.*, the food stuffs.

These substances are divided into:

I. Organic, of which we have two subdivisions.
  1. Nitrogenous bodies.
     Albumens and albumenoids.
  2. Non-nitrogenous bodies.
     Carbohydrates and fats.

II. Inorganic, two subdivisions.
  1. Water.
  2. Salts.

Our domestic carnivora obtain their nourishment from both animal and vegetable matter.

To obtain the best possible results in dog-breeding, it is necessary that their food contain all of these classes of foods; if deprived of any one of these, the animal will die sooner or later.

As the conditions which cause quickening or slowing of the metabolism are brought into play, so is the quantity of food required increased or diminished.

*Diet for Pregnant Bitches*—Is of that class best suited for the wants of both mother and young; should be pure, fresh and highly nutritious, and not of a kind to induce plethora or the laying on of flesh, together with a good supply of pure water and good hygienic surroundings. After parturition, a diet abundant in nutritive properties, as eggs, milk, bread and milk, broths and gruel. Fatty foods slow metabolism, while nitrogenous foods quicken it. A diet rich in carbohydrates will give rise to a condition of fat formation.

*For the Young.*—The proper diet for the young is its mother's milk. This is the food designed by nature for the young, and cannot be improved upon. Sometime, however, the mother may not have sufficient milk to supply her large litter; here the use of an artificial food becomes necessary. Parvin says "a mixed nursing is better than a whole diet of artificial food;" which means, allow the puppy to get a reasonable amount from the mother, and make up the deficiency with artificial food.

(Mr. Campbell here discussed to some length the diseases of the bitch, and the manner in which these diseases, together with the medicines used to produce their relief, affected the supply and quality of her milk.)

*Artificial Foods.*—The artificial food par excellence for old and young is milk. It contains proteids, fats, carbohydrates and water, and in such a condition that the most sensitive digestive tract can assimilate it. After puppies are weaned, cow's milk can be substituted for the mother's, and to this we add porridge, bread, etc., as he gets older. When powers of digestion increase, the milk can be substituted by broth, soups, etc., to a certain extent; also an occasional bone to pick will be a source of amusement, as well as help him cut his teeth and strengthen his jaws. A course of milk diet long continued will favor the growth and development of entozoa. This should receive careful attention.

*For the Adult.*—Spratt's biscuits are an excellent preparation, combining nearly all the properties of a perfect food, but they become rather expensive to an individual who keeps a large kennel. Where meat is fed, it should always be cooked, and thus destroy all the germs and parasites it may contain. In vigorous animals a little raw meat once in a while will do no harm, provided that the dog has plenty of exercise. Man's interference by artificial selection and domestication have wrought many changes in the dog. Every generation has placed him further and further from his natural condition, and no organ shows the effects of these changes more than the alimentary tract; very little upsets its workings. A good diet is boiled horseflesh, finely divided and mixed with equal parts of some starchy elements, such as bread, biscuit, etc., the whole made into a porridge with the liquor from the pot in which the horseflesh has been boiled. This diet is cheap and palatable, and could be fed advantageously to a large kennel.

*Diet in Disease.*—Here we have a departure from the normal condition. A diet which will produce the greatest possible good with the least possible harm to the system is indicated. We must, however, take the following precautions: 1. The food must not be contaminated by the presence of any germ. 2. We must not give a stimulating diet where the system is not strong enough to bear it. 3. When we have great depression, we must not overload the stomach with worthless substances, which in health are useless and doubly so in disease. Derangements of the digestive tract may be brought about by either overfeeding or not giving the dog enough of good nutritious diet. The effect of this is often shown in the skin. We are told "as the tongue to man so is the skin to the dog in indicating the condition of the ailmentary tract." This is essentially true, for by a system of overfeeding, the skin will become eczematous in character, and a large variety of serious complications result. Distention of the stomach by gas may cause irregular breathing and heart action, and if the distention be due to gas, it may proceed until rupture of the organ takes place. Emphysema of the lungs is probably due to this distention.

*Vomiting*—Diarrhœa and constipation may all be referable to the quality and quantity of food, etc. In a very excellent paper by Pasteur, it was remarked that bileary calculi were due in a great measure to excess of food and lack of exercise. Mr. Campbell concluded after pointing out minutely the value of the proper use of diet in various febrile conditions of the system, and also of the abuse that this valuable therapeutic agent was subjected to. Both Mr. Brainard's report and Mr. Campbell's essay were favorably commented upon by the President, and after each had been well discussed the meeting adjourned.

